# The Motivational Power of the Happy Face

**DOI:** 10.3390/brainsci9010006

**Published:** 2019-01-07

**Authors:** Jana Nikitin, Alexandra M. Freund

**Affiliations:** 1 Faculty of Psychology, Research Group Personality and Developmental Psychology, University of Basel, 4055 Basel, Switzerland; 2 Department of Psychology, University Research Priority Program Dynamics of Healthy Aging, University of Zurich, 8050 Zurich, Switzerland; freund@psychologie.uzh.ch

**Keywords:** emotional faces, approach, avoidance, motivation, reaction times

## Abstract

People who are cheerful have better social relationships. This might be the case because happy faces communicate an invitation to interact. Thus, happy faces might have a strong motivational effect on others. We tested this hypothesis in a set of four studies. Study 1 (*N* = 94) showed that approach reactions to happy faces are faster than other reactions to happy or angry faces. Study 2 (*N* = 99) found the same effect when comparing reactions to happy faces with reactions to disgusted faces. Supporting the notion that this effect is related to motivation, habitual social approach motivation intensified the motivational effect of happy faces (Study 3, *N* = 82). Finally, Study 4 (*N* = 40) showed that the reaction-time asymmetry does not hold for categorization tasks without approach and avoidance movements. These studies demonstrate that happy faces have a strong motivational power. They seem to activate approach reactions more strongly than angry or disgusted faces activate avoidance reactions.

## 1. Introduction

People who smile are rated more favorably by others and interactions with them are expected to be rewarding [1]. This might be one reason why cheerful people are happier [1], have a lower divorce rate [2], and live longer [3] than less cheerful people. Starting with the functional view of emotions by Darwin [4], it has long been recognized that emotions have an interpersonal function. People who smile express positive social intentions that are essential for the creation and maintenance of social bonds [5]. Smiling face and its frequent acoustic concomitant, laughter, are associated with bonding, agreement, and affection (for a summary, see Reference [6]). A happy face signals positive emotions, as well as attachment availability, care, support, and credibility [7,8,9]. Recently, Tamir and Hughes [10] argued that positive social signals such as smiling faces not only serve ultimate goals (e.g., forming strong bonds) but they are also rewarding in and off themselves. According to Tamir and Hughes, this proximal value of positive social signals forms the foundation on which the complexities of human sociality are built. In line with this hypothesis, Yang and Urminsky [11] demonstrated that anticipated positive affective reactions of social partners powerfully shape peoples’ behavior. In other words, people are highly motivated to evoke smile and happiness in others. In Yang and Urminsky’s studies, participants decided which gift they gave to a person. Participants often chose to forgo satisfaction-maximizing gifts in favor of gifts that maximized anticipated positive emotional reactions in the perceiver. These results confirmed the author’s “smile-seeking” hypothesis that peoples’ desire to induce, approach, and enjoy others’ spontaneous displays of affective reactions is innately valuable and rewarding, and consequently, a powerful motivator in social contexts (see also References [12,13,14,15]).

Similar conclusions come from neuropsychological research that link the reward areas of the basal ganglia with the perception of smiling faces [16]. The reward circuitry in the basal ganglia, in turn, is associated with what Davidson [17] termed *pre-goal attainment positive affect*. In Davidson’s view, activation in subcortical reward centers supports an organism’s approach toward an appetitive goal. It seems that the activation of the reward system by a perceived smile serves appetitive behavior. People who smile show signals of prosocial intentions [18]. In most—if not all—cases, approaching positive social stimuli is adaptive [19]. Given that approaching a happy person implies approaching safety and the possibility for affiliation, others might follow this invitation. In other words, a smiling face should motivate others to approach [20,21,22]. In fact, previous studies have demonstrated that people show approach behavioral tendencies towards smiling faces [20,21,23,24,25]. Although it has been argued repeatedly that these approach tendencies to happy faces have motivational underpinnings [10,15], to our knowledge the motivation hypothesis has not been directly tested. The present research aims at providing support for the motivation hypothesis by comparing the motivational effect of happy faces to facial expressions of negative emotions (anger, disgust). We assumed that approach behavior to happy faces is the default reaction. 

Approaching positive social stimuli such as happy faces should be generally adaptive. In contrast, it is less clear what the most adaptive reaction to negative social stimuli might be. For example, faces expressing anger communicate aggressive tendencies, and thus, possible threat [26,27]. In some cases of encountering anger, approach behavior might be most appropriate (e.g., when fighting against an aggressor is important for one’s social standing), but in other cases avoidance behavior might be more adaptive (e.g., when a fight might escalate aggression and likely leads to social or physical losses). Thus, angry facial expressions might not automatically elicit approach or avoidance behavior. Mineka [28] suggested that behavioral responses to (social) threat are slowed down in order to avoid erroneous decisions that could have detrimental consequences for the individual. Given the difficulties in deciding how to react best when confronted with anger, slowing down the reaction might give the person time to select the best solution in a given situation. In other words, there might be no clear default reaction to the display of anger. Indirect support for this assumption comes from a study by Roelofs, Hagenaars, and Stins [29]. Using a stabilometric force platform assessing the amount of spontaneous body sway, Roelofs and colleagues found reduced behavioral reaction when participants viewed angry compared to neutral and happy faces (for similar results, see Reference [25]). In addition, a recent meta-analysis [23] of 29 studies with 81 effects sizes found small- to medium-sized effects for the compatibility of different positive and negative stimuli (faces, pictures, and words) in the approach and avoidance tendencies, respectively [30]. Interestingly, however, there was a tendency of facial expressions to be less effective in initiating approach or avoidance behavior than all other stimuli. Particularly with respect to reactions to negative facial expressions such as anger, the results are mixed. Although there is good evidence that angry faces can be detected very quickly [31], they seem to be ambiguous with regard to the action tendencies they evoke. Some studies found congruent effects of angry faces and avoidance (compared to approach) behaviors [20,22,32], but other studies failed to detect any behavioral tendencies in reaction to angry faces [21,25,30,33,34].

In summary, we hypothesize that approach of happy faces is the default reaction, whereas there is no such default reaction to negative facial expressions. We test this hypothesis using a very basic motivated behavioral tendency to approach or avoid [35], measuring reaction times of arm movements [30]. The principle of this reaction-time task is that participants’ response speed to approach or avoid is affected by the compatibility between the response and the valence of the stimuli [36]. We start by testing the robustness of approach reactions to happy faces compared to reactions to negative facial expressions in Studies 1 and 2. We compare reactions towards happy faces with reactions to faces expressing anger (Study 1) and disgust (Study 2). We expect that participants react faster with approach than avoidance to happy faces and that the speed of approach and avoidance reactions to negative facial expressions does not significantly differ. Studies 3 and 4 were designed to test the motivational hypothesis more directly. Concretely, we test whether habitual approach motivation accelerates approach reactions to happy faces (Study 3) and we rule out that the reaction-time effects of happy faces are just categorization effects (Study 4).

## 2. Study 1

Study 1 tested the hypothesis that approach reactions to happy faces are faster than avoidance reactions to happy faces and that there is no clear reaction tendency to angry faces. To test this hypothesis, we used a distance-regulation paradigm. In this paradigm, approach and avoidance movements are associated with moving a manikin representing the self towards or away from emotional faces (for a similar approach, see Reference [37]). We used the manikin task instead of using arm flexion/arm extension as often used in the approach-avoidance task (AAT [30,38]) because the manikin approach is less ambiguous with respect to the reference point than the arm-flexion/arm-extension approach [32,39]. Arm flexion can be a movement towards oneself (indicating approach behavior) but also away from a stimulus (indicating avoidance behavior). Similarly, arm extension can be a movement away from oneself but also towards a stimulus. In the manikin task, the movements are unambiguously associated to approach and avoidance, respectively. In addition, the manikin task is more sensitive to valence of a stimulus (such as happy and angry faces) than the arm-flexion/arm-extension task [40].

### 2.1. Method

This research was conducted with the ethical guidelines of the University of Zurich; all studies were considered exempt from formal ethical review.

#### 2.1.1. Participants

Students of the University of Zurich were recruited within a larger project investigating social behavior in a transition from parental home to a shared apartment (see References [41,42,43]). Participants first completed an online questionnaire at home assessing sociodemographic variables and variables not relevant for the present research. Later, participants were invited to the lab for the reaction-time part of the study. They were informed that they would react to pictures of faces of different facial expressions. The sample consisted of 94 participants (82% female, age *M* = 23.93, *SD* = 4.33 years; socio-demographic data of 20 participants could not be assigned because of a wrong personal code, that means these participants created a different personal code for the online questionnaire assessing the sociodemographic information and for the reaction-time part of the study, so that their sociodemographic information could not be assigned to their reaction-time data (Excluding these participants from the main analysis did not change the results: we found a main effect of facial expression (*F*(1, 72) = 53.20, *p* < 0.001, η_p_^2^ = 0.43), a main effect of movement direction (*F*(1, 22) = 26.87, *p* < 0.001, η_p_^2^ = 0.27), and an expression × movement interaction, (*F*(1, 72) = 62.62, *p* < 0.001, η_p_^2^ = 0.47). No other effects were statistically significant (*p*s ≥ 0.12).). The majority of the participants (55.5%) was in a stable relationship or married, 44.6% were single. We included only participants with good knowledge of the German language. There were no other inclusion or exclusion criteria. Participants were recruited via emails sent from the university administration, via announcements on a university-wide job portal, and via flyers that were distributed all over the university. Participants gave written informed consent for participation. After participation, they were debriefed (i.e., they received a written document shortly explaining the research questions and hypotheses of the study) and received 20 Swiss francs (approximately 17 €) or extra course credit. 

#### 2.1.2. Stimuli and Procedure

Facial stimuli were chosen from the Lifespan Database of Adult Emotional Facial Stimuli [44]. We selected pictures of 110 models (27 young males, 28 young females, 28 middle-aged males, and 27 middle-aged females). One picture of each model clearly expressed happiness or anger, respectively. In order to delete peripheral information (such as hairdo, ears, neck) and to standardize the different models as much as possible, pictures were cut vertically from the hairline to the chin and horizontally at the cheekbones. Consequently, the picture width and length varied from 3.95” to 4.70”. For the highest possible uniformity, all pictures were gray scaled. We used the program DirectRT [45] for stimulus presentation, timing, and data collection. 

**Figure 1:**
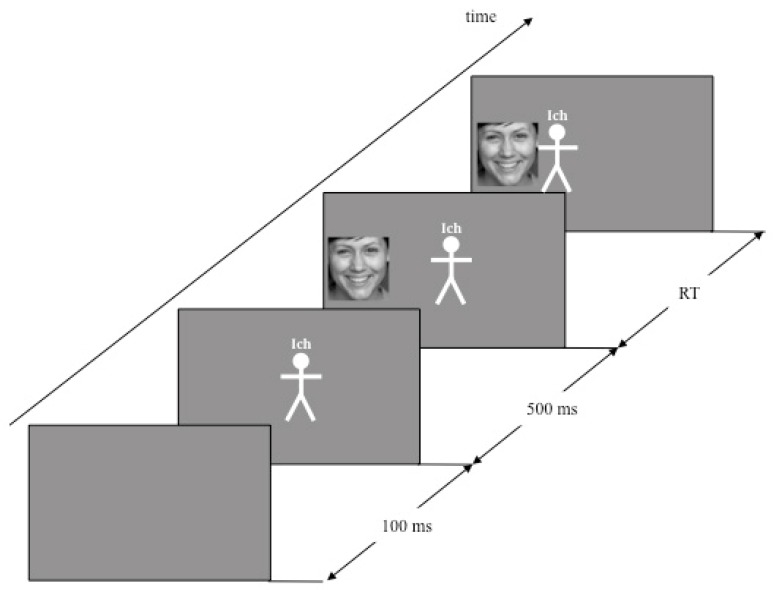
Exemplary schematic presentation of a congruent trial (“Ich” = “I”) from Study 1, 2, and 3.

An experimental trial started with a blank screen presented for 100 ms and was followed by a presentation of a small white manikin on a gray background in the middle of the screen (the procedure for an exemplary trial is shown in Figure 1). Participants were told that the manikin represents them, indicated by the superscription “I” (in German “Ich”) over its head. After another 500 ms, a picture of a happy or an angry face appeared randomly on the left or the right of the manikin. In half of the trials, the task was to move the manikin as fast as possible towards happy face and away from angry face (congruent trials). In the other half of the trials, the movement was reversed (incongruent trials). The trials were organized in four blocks, with alternating congruent and incongruent trials. Approximately half of the participants (*n* = 45) started with the congruent, and half (*n* = 49) with the incongruent condition. Half of the happy faces appeared on the left side, half of them on the right side. Half of them appeared in the congruent, half in the incongruent condition. The same was true for angry faces. The order of the presentation of the stimuli and their placement were randomized across participants. For the movements, participants used a joystick that was fixated on a board in the middle of the table. The manikin moved synchronously with the joystick movement.

### 2.2. Results and Dicussion

From a total of 20,680 trials, we excluded trials with incorrect responses, trials with reaction times above 1500 ms or below 200 ms, as well as responses that were more than two standard deviations above or below each participant’s mean response latency. Overall, 15.1% trials were excluded. There were 17,561 trials remaining in the data file. The amount of the remaining trials did not differ significantly between the four conditions, χ^2^(3, 17,561) = 1.28, *p* = 0.73. We ran all analyses with log-transformed reaction times to correct for skewness in the distribution.

**Figure 2:**
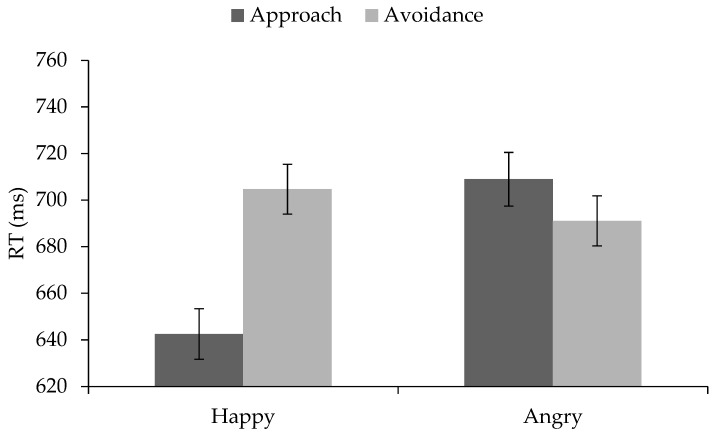
Mean reaction times (RTs) to happy and angry faces from Study 1. Error bars represent standard error of the mean.

We ran a 2 × 2 × 2 mixed-design ANOVA with facial expression (happy vs. angry) and movement direction (approach vs. avoidance) as within-participants factors and block order (congruent first vs. incongruent first) as a between-participants factor. The analysis revealed a main effect of facial expression (*F*(1, 92) = 80.71, *p* < 0.001, η_p_^2^ = 0.47) and a main effect of movement direction (*F*(1, 92) = 71.87, *p* < 0.001, η_p_^2^ = 0.44). People reacted faster to happy (*M* = 673.60 ms, *SD* = 102.76 ms) than to angry faces (*M* = 699.99 ms, *SD* = 101.23 ms) and faster with approach (*M* = 675.72 ms, *SD* = 102.03 ms) than with avoidance (*M* = 697.86 ms, *SD* = 101.58 ms). However, and in line with the hypotheses, this main effect was qualified by a significant expression × movement interaction, *F*(1, 92) = 37.57, *p* < 0.001, η_p_^2^ = 0.29 (There was neither a main effect of gender on the reaction times (*p* = 0.42), nor did gender moderate the effect of facial expression (*p* = 0.27), movement (*p* = 0.65), condition (*p* = 0.25), or the expression × movement interaction (*p* = 0.13) on the reaction times). As shown in Figure 2, approach reactions to happy faces were faster than avoidance reactions to happy faces (*t*(93) = −9.58, *p* < 0.001, *d* = 1.99), they were faster than avoidance reactions to angry faces (*t*(93) = −11.12, *p* < 0.001, *d* = 2.31), and also faster than approach reactions to angry faces (*t*(98) = −9.17, *p* < 0.001, *d* = 1.90). Avoidance reactions to happy faces were not significantly different from avoidance reactions to angry faces (*t*(93) = 1.46, *p* = 0.15) and also not different from approach reactions to angry faces (*t*(93) = −1.05, *p* = 0.30). Avoidance reactions to angry faces were marginally faster than approach reactions to angry faces (*t*(93) = −1.90, *p* = 0.06, *d* = 0.39). The main effect of block order (*F*(1, 92) = 2.82, *p* = 0.10) as well as the interaction between block order, facial expression, and movement were not statistically significant (*F* < 1).

In sum, then, results of Study 1 supported the hypothesis of faster approach reactions to happy faces compared to all other conditions. When participants were instructed to approach happy faces, they were faster than when they were instructed to avoid happy faces or to approach or avoid angry faces. These results are in line with the assumption that approaching happy faces is the default—and therefore the fastest—reaction and support previous findings on clear (default) approach tendencies towards happy faces [20,21,23,24,25]. Our results also indicate that there does not seem to be a strong default reaction to angry faces, although people are by trend faster when avoiding angry faces than when approaching them. Thus, our results are in line with other studies that failed to find clear (unambiguous) behavioral tendencies to angry faces [21,25,30,33,34]. 

## 3. Study 2

Study 2 aimed at replicating the results of Study 1. In addition, Study 2 used different negative facial expressions to rule out the possibility that the reaction-time effects of Study 1 are driven by the comparison of happy and angry faces. We chose faces expressing disgust. Different to angry faces, disgusted faces do not express a direct threat towards the perceiver; however, they express a clearly negative emotion [46]. Thus, we expect that—similarly to angry faces—there is no default reaction to faces expressing disgust. In line with this hypothesis, Seidel and colleagues [21] found no significant tendency to approach or avoid faces expressing disgust.

### 3.1. Method

#### 3.1.1. Participants

We recruited students of the University of Zurich to participate in a lab study entitled “Reactions to Faces.” Participants first completed an online questionnaire at home assessing sociodemographic variables and variables not relevant for the present research. Later, participants were invited to the lab for the reaction-time part of the study. They were informed that they would react to pictures of faces of different facial expressions. The sample consisted of 99 participants (77% female; age *M* = 24.00, *SD* = 3.00 years; socio-demographic data of three participants could not be assigned because of a wrong personal code). The majority of the participants (67.7%) were single, 32.2% was in a stable relationship or married. We included only participants with good knowledge of the German language. There were no other inclusion or exclusion criteria. Participants were recruited via announcements on a university-wide job portal and via flyers that were distributed all over the university. Participants gave written informed consent for participation. After participation, they were debriefed (i.e., they received a written document shortly explaining the research questions and hypotheses of the study) and received 20 Swiss francs (approximately 17 €) or extra course credit.

#### 3.1.2. Stimuli and Procedure

We selected pictures of the same 110 young models as in Study 1. One picture of each model clearly expressed happiness or disgust, respectively. The procedure was the same as in Study 1. Approximately half of the participants (*n* = 49) started with the congruent, half (*n* = 50) with the incongruent condition.

### 3.2. Results and Dicussion

From a total of 21,780 facial-expression trials, we excluded 8.7% trials using the same criteria as in Study 1. There were 19,888 trials remaining in the data file. The amount of the excluded trials did not differ significantly between the four conditions, χ^2^(3, *N* = 21,780) = 2.06, *p* = 0.56. 

**Figure 3:**
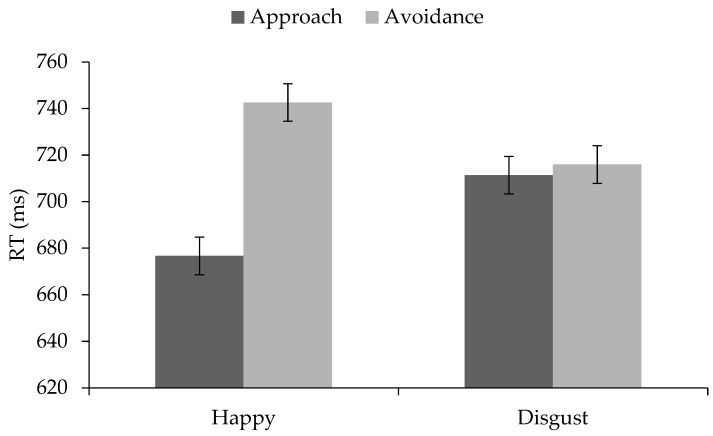
Mean reaction times (RTs) to happy and disgusted faces from Study 2. Error bars represent standard error of the mean.

We ran a 2 × 2 × 2 mixed-design ANOVA with facial expression (happy vs. disgusted) and movement direction (approach vs. avoidance) as within-participants factors and block order (congruent first vs. incongruent first) as a between-participants factor. The analysis revealed a main effect of facial expression (*F*(1, 97) = 5.19, *p* = 0.03, η_p_^2^ = 0.05) and a main effect of movement direction (*F*(1, 97) = 54.69, *p* < 0.001, η_p_^2^ = 0.36). People reacted slightly faster to happy (*M* = 709.61 ms, *SD* = 77.44 ms) than to disgusted faces (*M* = 713.63 ms, *SD* = 74.53 ms) and faster with approach (*M* = 694.0 ms, *SD* = 75.38 ms) than with avoidance (*M* = 729.24 ms, *SD* = 77.30 ms). However, and in line with the hypotheses, this main effect was qualified by a significant expression × movement interaction (*F*(1, 97) = 151.39, *p* < 0.001, η_p_^2^ = 0.61). As shown in Figure 3, approach reactions to happy faces were faster than avoidance reactions to happy faces (*t*(98) = −13.07, *p* < 0.001, *d* = 2.64), they were faster than avoidance reactions to disgusted faces (*t*(98) = −7.06, *p* < 0.001, *d* = 1.43) and also faster than approach reactions to disgusted faces (*t*(98) = −10.21, *p* < 0.001, *d* = 2.06). In contrast, avoidance reactions to happy faces were slower than avoidance reactions to disgusted faces (*t*(98) = 5.28, *p* < 0.001, *d* = 1.07) and also slower than approach reactions to disgusted faces (*t*(98) = 8.44, *p* < 0.001, *d* = 1.71). Approach and avoidance reactions to disgusted faces did not significantly differ, *t*(98) = 1.51, *p* = 0.14. The main effect of block order (*F* < 1) as well as the interaction between block order, facial expression, and movement were not statistically significant, *F*(1, 97) = 1.04, *p* = 0.31. (Although there was a main effect of gender on the reaction times (*F*(1, 92) = 4.13, *p* = 0.045), suggesting that males reacted faster (*M* = 684.33, *SD* = 65.21) than females (*M* = 720.34, *SD* = 75.30), gender did not moderate the effect of facial expression (*p* = 0.58), movement (*p* = 0.68), condition (*p* = 0.73), or the expression × movement interaction (*p* = 0.71) on the reaction times.)

In sum, then, Study 2 replicated the finding of Study 1 that approach reactions to happy faces are faster than any other reaction. Importantly, Study 2 showed that this effect is not limited to the comparison of happy and angry faces but extends to the comparison of happy and disgusted faces. (To test whether approach and avoidance reactions to emotional faces differed between Study 1 and 2, we computed difference scores between the log-transformed reaction times of avoidance reactions minus approach reactions to happy, angry, and disgusted faces. A mixed-design ANOVA tested whether the difference scores differed dependent on the interaction between Valence (positive vs. negative facial expression) and Study (Study 1 vs. Study 2). There was a main effect of Study (*F*(1,191) = 7.12, *p* = 0.008, η_p_^2^ = 0.04) and a main effect of Valence (*F*(1,191) = 83.70, *p* < 0.001, η_p_^2^ = 0.04). The difference between approach and avoidance movements was generally larger in Study 2 (*M* = 35.24 ms, *SD* = 29.58 ms) than in Study 1 (*M* = 22.14 ms, *SD* = 28.72 ms) and the difference was generally larger for happy (*M* = 64.09 ms, *SD* = 61.26 ms) than for angry/disgusted faces (*M* = −6.37 ms, *SD* = 66.76 ms). Importantly, there was no significant valence × study interaction, *F*(1,191) = 1.91, *p* = 0.17, suggesting that the pattern of responses to happy vs. angry and happy vs. disgusted faces is comparable in both studies.)

## 4. Study 3

Study 3 tested the hypothesis that the faster approach reactions to happy faces have motivational roots. To that end, Study 3 investigated whether habitual social approach motivation, i.e., the general tendency to approach positive social outcomes [47], intensifies the approach effect of happy faces. This should be the case because habitual social approach motivation energizes and directs behavior towards positive social information such as smiling faces (for summaries, see References [48,49]). In fact, habitual social approach motivation enhances the exposure to positive social events in friendships [50], active approach behavior in social interactions with strangers [51], and extraverted behavior in interactions with roommates [41], suggesting appetitive behavioral tendencies towards social rewards. As behavioral tendencies of habitual motives are assumed to be embodied and automatic [15,52,53], habitual social approach motivation should be associated with very basic behavioral approach tendencies towards happy faces as measured in the present research. 

Study 3 again used happy and angry faces. As Study 1 and 2 demonstrated that the reaction-time differences between reactions to happy and negative facial expressions are driven by faster approach reactions to happy faces than other reactions to angry or disgusted faces, Study 3 used only approach movements to happy faces and avoidance movements to angry faces. Our hypothesis was that the higher the habitual social approach motivation is, the faster are approach reactions to happy faces. As habitual approach motivation regulates responses to positive but not to negative social information (e.g., [54]), we did not expect habitual approach motivation to be associated with reaction times to angry faces.

### 4.1. Method

#### 4.1.1. Participants

As in Study 2, we recruited students of the University of Zurich to participate in a lab study entitled “Reactions to Faces.” Participants first completed an online questionnaire at home assessing sociodemographic variables, habitual social approach motivation, and variables not relevant for the present research. Later, participants were invited to the lab for the reaction-time part of the study. They were informed that they would react to pictures of faces of different facial expressions. The sample consisted of 82 participants (73% females, age *M* = 26.77, *SD* = 6.29 years). Approximately half of the participants (48.8%) were single, 50.0% was in a stable relationship or married, and one person was divorced. We included only participants with good knowledge of the German language. There were no other inclusion or exclusion criteria. Participants were recruited via announcements on a university-wide job portal and via flyers that were distributed all over the university. The study was run in the laboratories of the University of Zurich. Participants gave written informed consent for participation. After participation, they were debriefed (i.e., they received a written document shortly explaining the research questions and hypotheses of the study) and received 20 Swiss francs (approximately 17 €) or extra course credit. 

#### 4.1.2. Stimuli and Procedure

We used the same stimuli and the identical procedure as in Study 1 but included only the happy-approach and angry-avoidance conditions.

#### 4.1.3. Assessment of Habitual Social Approach Motivation

The Affiliation Tendency Scale [55], German version in Reference [56], was used to assess habitual social approach motivation. This scale consists of 25 self-descriptive items (e.g., “I like to make as many friends as I can”). Responses were given on a rating scale ranging from 0 (strongly disagree) to 6 (strongly agree). The internal consistency of the scale was Cronbach’s *α* = 0.74 (*M* = 3.88, *SD* = 0.55).

### 4.2. Results and Dicussion

From a total of 18,040 trials, we excluded 5.96% of the trials using the same exclusion criteria as in Study 1 and 2. There were 16,966 trials remaining in the data file. The number of remaining trials did not significantly differ between the two conditions, χ^2^(1, *N* = 16,966) = 0.44, *p* = 0.51. All analyses were run with log-transformed reaction times to correct for skewness in the distribution.

**Figure 4:**
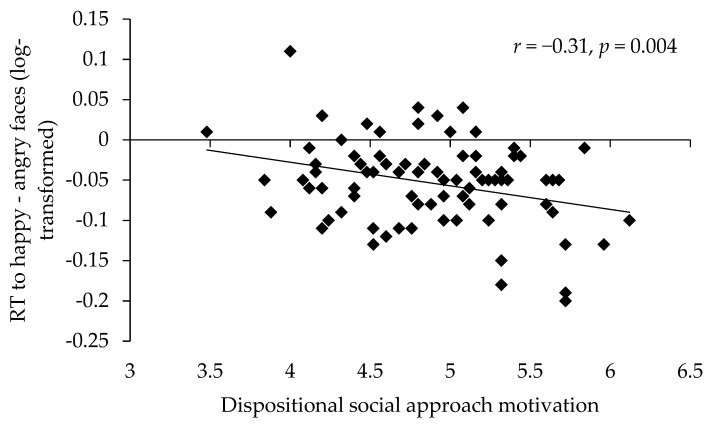
Bivariate correlation between habitual social approach motivation and the reaction-time difference between approach reactions to happy and avoidance reactions to angry faces (Study 3). Positive values represent slower reactions to happy than to angry faces, negative values represent faster reactions to happy than to angry faces.

The average reaction times to happy faces (*M* = 625.62 ms, *SD* = 87.13 ms) were significantly faster than the average reaction times to angry faces (*M* = 652.64 ms, *SD* = 82.78 ms), *t*(81) = −9.25, *p* < 0.001, *d* = 2.06. Bivariate correlations of reaction times and habitual social approach motivation supported the motivational hypothesis: The higher the habitual social approach motivation was, the faster were approach reactions to happy faces (*r* = −0.28, *p* = 0.01). The correlation between social motivation and avoidance reactions to angry faces was not statistically significant (*r* = −0.14, *p* = 0.22). Importantly, social approach motivation was correlated with the difference in reaction times to happy and angry faces (*r* = −0.31, *p* = 0.004). The higher the social approach motivation was, the faster participants reacted to happy compared to angry faces (see Figure 4). (There was neither a main effect of gender on the reaction times (*p* = 0.18), nor did gender moderate the effect of facial expression (*p* = 0.52) on the reaction times. There were also no significant moderation effects of gender on the associations between habitual approach motivation and the reaction times to happy and angry faces and their difference (all *p*s ≥ 0.16).)

Taken together, Study 3 supported the hypothesis that habitual social motivation accelerates approach reactions to happy faces. Happy faces seem to have a particular motivational power for people who are generally highly motivated to approach positive social outcomes, supporting the motivational explanation of approach reactions to happy faces.

## 5. Study 4

Study 4 was conducted to ensure that the reaction-time effects of happy faces are not just categorization effects but are motivational in nature. In other words, we wanted to rule out that participants react faster to happy compared to angry faces because happy faces are faster recognized or processed [57]. This would be the case when participants would react faster to happy than to angry faces even when they do not execute any approach behavior. To test this possibility, Study 4 used the same stimuli as Study 1 and 3 (i.e., happy and angry faces) but instead of reacting with approach and avoidance, the task was to categorize the facial expressions by pressing a response key. We expected no significant differences in the reaction times between reactions to happy and angry faces as these reactions were not associated with approach or avoidance behavior. In addition, we tested whether habitual social approach motivation affects the reaction times. We expected that because the task is not related to approach reactions, habitual social approach motivation would not be associated with the reaction times.

### 5.1. Method

#### 5.1.1. Participants

We recruited students of the University of Zurich to participate in a lab study entitled “Reactions to Faces.” Participants first completed an online questionnaire at home assessing sociodemographic variables, habitual social approach motivation, and variables not relevant for the present research. Later, participants were invited to the lab for the reaction-time part of the study. They were informed that they would react to pictures of faces of different facial expressions. The sample consisted of *N* = 40 young adults (73% female, age *M* = 25.30, *SD* = 4.42 years). The majority of the participants (62.5%) were single, 37.5% was in a stable relationship or married. We included only participants with good knowledge of the German language. There were no other inclusion or exclusion criteria. Participants were recruited via announcements on a university-wide job portal and via flyers that were distributed all over the university. Participants gave written informed consent for participation. After participation, they were debriefed and received 20 Swiss francs (approximately 17 €) or extra course credit. 

#### 5.1.2. Stimuli and Procedure

We used the same stimuli as in Study 1 and 3. An experimental trial started with a blank screen presented for 100 ms and was followed by a presentation of a picture of a happy or an angry face in the middle of the screen. The task was to press a choice button with the left or right index finger, respectively. Index fingers were placed on the buttons throughout the experiment. Half of the participants (*n* = 20) pressed the left key for happy and the right key for angry faces, the other half (*n* = 20) responded in the reversed way.

#### 5.1.3. Assessment of Habitual Social Approach Motivation

The Affiliation Tendency Scale was again used to assess habitual social approach motivation. The internal consistency of the scale was Cronbach’s *α* = 0.74 (*M* = 3.71, *SD* = 0.57).

### 5.2. Results and Dicussion

From a total of 8800 facial-expression trials, we excluded 4.2% of the trials using the same exclusion criteria as in the previous studies. There were 8428 trials remaining in the data file. The amount of remaining trials in the two conditions did not significantly differ, χ^2^(1, *N* = 8428) = 0.01, *p* = 0.91. All analyses were run with log-transformed reaction times to correct for skewness in the distribution.

We ran a mixed-design ANOVA with facial expression (happy vs. angry) as a within-participants factor and the response-key allocation (positive left/negative right vs. positive right/negative left) as a between-participants factor. There was no main effect of facial expression (*F* < 1). Reaction times to happy faces (*M* = 495.94 ms, *SD* = 74.46 ms) and reaction times to angry faces (*M* = 492.97 ms, *SD* = 74.61 ms) did not differ significantly. There was also no effect of the response-key allocation (*F* < 1) or an interaction effect of facial expression and response-key allocation, *F*(1, 38) = 1.55, *p* = 0.22. In addition, habitual social approach motivation did not correlate with reaction times to happy (*r* = −0.01, *p* = 0.97) or angry faces (*r* = −0.06, *p* = 0.71). There was also no significant correlation between social approach motivation and the difference in the reactions to happy and angry faces (*r* = 0.14, *p* = 0.38). (There was neither a main effect of gender on the reaction times (*p* = 0.17), nor did gender moderate the effect of facial expression (*p* = 0.66), response-key allocation (*p* = 0.18), or their interaction (*p* = 0.91) on the reaction times. There were also no significant moderation effects (conducted with the programm PROCESS, 91) of gender on the associations between habitual approach motivation and the reaction times to happy and angry faces and their difference (all *p*s ≥ 0.21).)

Study 4 further supported the notion that faster reactions to happy faces have motivational roots. Categorization of emotional faces without approach and avoidance movements did not reveal the reaction-time differences that we observed in Studies 1–3. In addition, habitual social approach motivation was not associated with the reaction times in Study 4. We maintain that this is the case because pressing a response key does not reflect an approach of the happy face. (Because it is problematic to accept a Null-hypothesis, we contrasted results of Study 4 with the previous three studies. To this end, we calculated difference scores between the log-transformed reaction times to angry/disgusted minus happy faces for all four studies (we used only the approach movements to happy and avoidance movements to angry/disgusted faces from Study 1 and 2) and compared them in a univariate ANOVA with Study (1–4) as a between-participants factor. There was a significant effect of the factor Study, *F*(3,311) = 19.63, *p* < 0.001, η_p_^2^ = 0.04. The post-hoc tests (LSD) revealed significant differences between Study 4 and all other three studies (all *p*s < 0.001). No other differences were statistically significant (all *p*s > 0.05). Thus, the differences between happy and angry/disgusted faces in Studies 1, 2, and 3 cannot be interpreted as a result of simple categorization.)

## 6. General Discussion

Since Harlow’s [58] classic studies and the advent of attachment theory [59,60], belongingness has been acknowledged as one of the most fundamental human motives [61]. The present studies applied this motivational hypothesis to study the reactions to emotional faces. Study 1 and 2 supported the robustness of approach reactions to happy faces. Approach reactions to happy faces were faster than any other reaction to happy, angry, or disgusted faces. Study 3 demonstrated that habitual social approach motivation intensifies the motivational power of happy faces. Importantly, results of Study 4 indicate that this effect might be specific to approach reactions. Simple categorization without approach did not reveal the happy-face advantage in reaction times, supporting the notion that the effect is driven by motivational factors. In the following, we discuss the results of the present studies and their theoretical and practical implications.

### 6.1. Happy Faces

In summary, people react very fast to happy faces with approach behavior. This is not only the result of the present studies but is supported without exception by previous research that investigated approach and avoidance reactions to happy faces [23]. This might be the case because approaching a happy person might often have benefits and rarely costs. Humans rely on other people, and therefore, have to approach them. The high survival value of other people is arguably the reason why belongingness is one of the most fundamental human needs and why we strive for positive social encounters most of the time [61]. A smile signals positive social intentions [5] and the possibility to satisfy our desire for belongingness. This might be the reason why a happy face has a strong motivational power to approach. This conclusion is most strongly supported by Study 3 of the present research, that showed a positive association between the strength of habitual social approach motivation and speed of approach reactions to happy faces, and Study 4, that showed no such differences in a categorization task. 

Further indirect support for this motivational interpretation comes from studies investigating patients with depression. For example, Derntl and colleagues [33] found less amygdala activation (indicating reduced reactivity in response to positive emotional stimuli; [62]) in patients with major depression compared to healthy controls during approach movements toward happy faces. Additionally, more pronounced depressive symptoms in the patient group were accompanied by lower levels of amygdala activation. As depressive symptoms are negatively linked to appetitive motivation [63], these results suggest that people with low levels of appetitive motivation are less sensitive to positive social signs (for similar results, see Reference [24]). Moreover, research on social anxiety shows that highly socially anxious persons show avoidance tendencies to smiling faces [30]. This might be the case because socially anxious persons do not expect benevolent intentions of their interaction partners [64], and thus avoid social interaction partners even when the interaction partners express positive emotions [65]. 

These findings do not only support the motivational explanation of approach of happy faces, they also demonstrate the boundaries of the fundamentality of these reactions. Although we assume that most people search positive social interactions most of the time, there are also exceptions. These exceptions are explained either by individual differences such as low appetitive motivation associated with depressive symptoms [24,33] or possibly negative evaluation of happy faces associated with social anxiety [30,65]. However, there are also situational factors that reduce approach tendencies to happy faces, particularly when the smiling face signals dominance, Schadenfreude, or shame and embarrassment [14,66,67]. This might be the case in situations that are hostile or competitive. In support of this hypothesis, Paulus and Wentura [22] found that smile in members of an outgroup (e.g., Middle-Eastern men for White-Caucasian participants) are associated with faster avoidance than approach reactions. The authors concluded that a smile of an outgroup member is evaluated more probably as dominance, arrogance or Schadenfreude rather than friendliness and cooperation, and thus lead to avoidance reactions (for similar results, see Reference [68]). Future research should investigate more systematically, in which situations and in whom smiling faces do (not) lead to strong approach tendencies. 

### 6.2. Negative Facial Expressions

An interaction with an angry person or a person expressing disgust is ambivalent. If someone signalizes anger directed to us, we might experience a conflict between the need to belong and the wish to avoid a negative social encounter. This might slow down our reactions. The same might be true for disgusted faces. Accordingly, studies investigating approach and avoidance tendencies towards angry (and disgusted) faces provide mixed results [21,25,30,33,34]. Even in studies that find faster avoidance than approach responses to angry faces (e.g., [20]), responses to anger expressions are typically slower than responses to other emotional expressions (see also [21]). The present studies support previous findings indicating that there is no default behavioral reaction to negative facial expressions such as anger or disgust. In contrast to negative words [37,69], negative attitude objects [35], negative auditory stimuli [70], negative affective pictures [71], or fear-related animals such as spiders [38], negative facial expressions do not seem to elicit a default avoidance reaction. Instead, negative facial expressions, and particularly angry facial expressions, seem to lead to slower reactions, irrespective of approach or avoidance [20,21]. 

This might be the case because angry facial expressions activate neural circuits involved in behavioral suppression [72], and thus lead to behavioral inhibition in the perceiver [25,29]. Such inhibition might reflect an orienting response during which we prepare an appropriate reaction. As Phaf and colleagues [23] put it, angry faces seem to require further interpretation to elicit either approach, when evoking anger in the perceiver (e.g., [34]), or avoidance, when evoking fear (e.g., [32]. Similarly, Seidel and colleagues [21] argued that disgust in facial expression can signal different messages that request either approach (e.g., food-offence disgust) or avoidance (e.g., individual-related disgust) [73]. As Winkielman and colleagues [15] discussed, the social environment is complex, and it demands context-appropriate responses. It is an interesting direction for future research to modulate context variables to test directly, which variables elicit approach and which avoidance behavior as a reaction to negative facial expressions [22].

### 6.3. Limitations and Future Directions

One limitation of the present studies is the artificial setting and task. Looking at still faces at a computer screen and reacting to them using a joystick bears no resemblance to real-life situations and carries no real-life relevance. A natural setting would provide more contextual information such as verbal and non-verbal behavior, the type of relationship, or whether the environment is safe or not. Such contextual information might be particularly helpful for deciding how to react when being confronted with a negative facial expression. Therefore, with more contextual information, the reaction to negative facial expressions might come as readily as the reaction to a happy face. On the other side, the context-free setting emphasizes the motivational power of happy faces. Even when there is no contextual information, people’s approach reactions to happy faces are faster than any other reaction to happy, angry, or disgusted faces.

Another critical point of our studies is that we cannot disentangle the effect of valence from the effect of arousal. Happy faces are lower in arousal than angry or disgusted faces (e.g., 77). Thus, people might react slower to angry and disgusted faces than to happy faces because angry and disgusted faces are more arousing. Contradicting this assumption, Robinson and colleagues [74] found a faster processing of highly arousing negative stimuli compared to negative stimuli low in arousal. Similarly, Lang [75] provided evidence that negative and highly arousing stimuli elicit behavioral responses with a larger amplitude than would be expected only on the basis of their valence. Thus, if arousal had any effect on the current findings, it should lead to faster reactions to angry or disgusted faces than to happy faces. In contrast, we find faster reactions to happy faces than to negative emotional expressions, speaking for the effect of valence rather than the effect of arousal.

Further, the present studies are based on samples with considerably more females than males, which can be partly explained by the fact that the studies were run in the labs of the department of psychology and many of the participants were psychology students who are, in majority, female. It is not clear whether the present findings can be generalized to other populations. Note, that we tested possible moderation effects of gender on the present findings and found no evidence for such moderation effects. We caution, however, that the male samples were too small to draw reliable conclusions from these additional analyses. In fact, there is some evidence for gender differences in social motivation: for example, women rate themselves as higher in warmth and empathy than men [76] and they are more other-oriented and more cautious than men [77]. This could explain some findings of the present research (e.g., faster approach reactions to happy faces and slower reactions to negative facial expressions). Although gender differences both in socially relevant (such as empathy; [78]) as well as general psychological factors [79] are relatively small, the present research should be nevertheless replicated in studies with more balanced gender distributions.

In the present studies, we did not measure the ambiguity of the reactions to angry faces directly but tested a possible consequence of this ambiguity, namely longer reaction times to angry as compared to happy faces. Further studies need to test the link between the difficulty to choose the appropriate reaction facing an angry person and reaction times directly. One possibility is to use the “mouse tracking” procedure that allows investigating the process of decision-making [80].

Finally, some studies using the categorization task found different results than Study 4 of the present research [57,81]. These studies found faster categorization of happy faces than of negative facial expressions. These results were explained by (1) a general bias of expecting a higher base rate of positive over negative events, including facial expressions [57]; (2) a match between one’s (positive) mood [82] and the encountering of a (positive) facial expression in others; and (3) an asymmetry between the number of positive (one: smiling face) and negative facial expressions of basic emotions (four: anger, sadness, disgust, fear; [57]). There are differences in the method of previous categorization studies and of the current Study 4 that might account for the different findings. First, we used a high number of models for the facial expressions so that each model was presented only two (Studies 3 and 4: happy and angry facial expression) or four times (Studies 1 and 2: happy and angry/disgusted facial expression in the approach and the avoidance condition). In contrast, previous categorization studies used a small number of models or even schematic facial expressions that were presented many times [57,81]. This could have led to learning effects that might be stronger with respect to happy than to negative facial expressions. In addition, previous categorization studies did not compare the findings from the categorization task with possible results of an approach-avoidance task. Thus, they cannot rule out additional acceleration of responses to happy faces when happy faces are associated with approach reactions. Future research is needed to test these possible differences directly.

## 7. Conclusions

The present research investigated how basic behavioral reactions might help us to understand the functionality of social behavior. Happy faces signal attachment availability and people react readily to this signal. By their motivational power, happy faces help to satisfy the need to belong of both the smiling person and the perceiver. Thus, an intensive smile seems to have an important interpersonal function. The present findings lead to several important conclusions within this framework. 

First, they show that the reaction-time advantage to happy compared to negative facial expressions is driven by motivational factors. Although it has been repeatedly found that people react faster to happy faces than to negative facial expressions, we still know very little about why this is the case. The present studies illustrate the importance of motivational factors in the happy-face behavioral advantage.

Second, the present studies indicate that there is no default behavioral reaction to negative facial expressions such as anger or disgust. In contrast to negative words [37,69], negative attitude objects [35], negative auditory stimuli [70], negative affective pictures [71], or fear-related animals such as spiders [38], negative facial expressions do not seem to elicit a default avoidance reaction (see also Reference [30]). Instead, negative facial expressions seem to lead to slower reactions, irrespective of approach or avoidance. This might be the case because negative facial expressions are associated with behavioral inhibition [29]. Such inhibition might reflect an orienting response during which we prepare an appropriate reaction.

Third, the present research provides support for the notion that intervention studies that are based on the approach-avoidance task might train motivation (and not simply associations between stimuli and reactions). Such interventions use for example the adoption of approach-type postures [83], execution of approach-type movements towards rewarding choices [84] or positive facial expressions [85], and avoidance-type movements that lead to more controlled (less impulsive) information processing [86]. One of these studies has also demonstrated that socially anxious participants who were trained to approach smiling faces displayed more social approach behaviors during a subsequent social interaction compared to participants in the control group [87]. The present research provides support for the motivational explanation of these trainings, and thus contributes to better understanding of the mechanisms that underlie such training effects.
